# Autophagy in neurodegeneration and aging

**DOI:** 10.18632/aging.101652

**Published:** 2018-11-14

**Authors:** Yasuo Miki, Janice L Holton, Koichi Wakabayashi

**Affiliations:** 1 the Queen Square Brain Bank for Neurological Disorders, Department of Molecular Neuroscience, UCL Institute of Neurology, London, UK; 2 Hirosaki University Graduate School of Medicine, Department of Neuropathology, Institute of Brain Science, Hirosaki, Japan

**Keywords:** autophagy, synucleinopathy, Parkinson’s disease, dementia with Lewy bodies, multiple system atrophy, α-synuclein

The histopathological hallmark of Parkinson’s disease (PD) and dementia with Lewy bodies (DLB) is the occurrence of insoluble fibrillary aggregates known as Lewy bodies, in which phosphorylated α-synuclein is a major component. Phosphorylated α-synuclein is also a constituent protein of glial cytoplasmic inclusions in multiple system atrophy (MSA). Thus, PD, DLB and MSA give rise to a disease concept of synucleinopathy. Macroautophagy (referred to as autophagy hereafter) is a highly conserved degradation pathway whereby cytosolic components as well as aberrant proteins are sequestered within double-membraned vesicles, known as autophagosomes. Autophagy consists of three processes: initiation of autophagy, formation of autophagosomes, and degradation of abnormal proteins after fusion with lysosomes. Each process is meticulously controlled and failure of any step of autophagy can lead to accumulation of abnormal proteins, including α-synuclein, in neurons, resulting in neuronal cell death [1].

Dysfunction of autophagy is now acknowledged as one of the features in several neurodegenerative disorders such as Alzheimer’s disease, amyotrophic lateral screlosis, Huntington’s disease and synucleinopathies. Indeed, in postmortem brains of patients with PD and DLB, upstream autophagosomal proteins (UNC-51-like kinase 1/2 (ULK1/2), Beclin1, vacuolar protein sorting-associated protein 34 (VPS34), and autophagy/beclin1 regulator 1 (AMBRA1)), which initiate autophagy and form autophagosomes, were found to be involved in Lewy body formation. Immunoblotting using fractionated brain lysates from DLB patients showed Beclin1 and VPS34 in soluble and insoluble smear bands of phosphorylated α-synuclein. Much stronger bands of ULK1 and ULK2 were found in the soluble fraction of DLB patients. We also investigated the alteration of autophagy in a cellular model of PD, demonstrating chronological upregulation of ULK1, ULK2, Beclin1 and VPS34 in response to accumulation of α-synuclein whereas in the brains of PD and DLB, increase of these upstream autophagosomal proteins was only partial [2]. Moreover, in surface plasmon resonance analysis, mutant α-synuclein had nine-times higher affinity for AMBRA1 compared with wild type α-synuclein, suggesting sequestering AMBRA1 from its original position [3]. Investigating the downstream part of the autophagy pathway, we were also able to demonstrate dysfunction indicated by alteration of microtubule-associated protein 1 light chain 3 (LC3) and γ-aminobutyric type A receptor associated proteins (GABARAPs), key regulators in this part of the autophagy pathway [4]. Taken together, our findings suggest dysregulation in various steps of autophagy in the pathogenesis of PD and DLB. However, because these results are based on in-vitro or postmortem studies, it has not been clarified whether impairment of autophagy represents a fundamental aspect of PD, or is the final stage of dysregulation resulting from a long neurodegenerative process. Therefore, in order to improve our understanding of altered autophagy in the early stage of PD, we then studied basal activity of autophagy using peripheral blood mononuclear cells (PBMCs) of patients with PD and control subjects. The whole-transcriptome assay revealed down-regulation of mRNAs for six core regulators of autophagy. Protein levels of ULK1, Beclin1 and AMBRA1 were increased with negative feedback on mRNA expression for these proteins in PD. There was significant correlation between these protein levels and α-synuclein in PBMCs. The expression level of the oligomeric form of α-synuclein in PBMCs paralleled the clinical severity and degeneration of cardiac sympathetic nerves in PD [5]. Collectively, our findings indicate that impairment of autophagy, which can occur at the various steps of autophagy, can occur during the disease process of PD and may precede the clinical manifestation of disease. Next, we explored the alteration of autophagy in postmortem brains of MSA. Although immunohistochemical and biochemical analyses revealed different patterns of impaired autophagy between Lewy body disease and MSA, both disorders shared dysfunction of the autophagosome closure process [4,6].

Dysfunction of protein degradation pathways is implicated in the formation mechanism of Lewy bodies and glial cytoplasmic inclusions. Kim et al. demonstrated that lysosomal degradation was impaired with aging, and genetic and pharmacological anti-aging manipulations alleviated propagation of α-synuclein in Caenorhabditis elegans models [7]. However, little is known about whether basal activity of autophagy could affect progression of disease among individuals with synucleinopathies, especially MSA. It would be of great interest to compare alteration of autophagy between MSA patients with short and prolonged disease durations, and with minimal and severe pathological change. Investigation of differences among individuals in the activity of autophagy might enable us to understand the variable severity of neurodegeneration in synucleinopathies, leading to the development of future therapeutic strategies to delay accumulation of abnormal α-synuclein.
